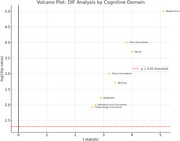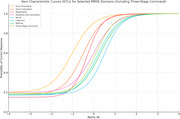# Influence of Depression on Cognitive Item Responses of the Korean version of MMSE: An Item Response Theory‐Based Analysis Controlling for Age and Education

**DOI:** 10.1002/alz70861_108289

**Published:** 2025-12-23

**Authors:** Sungman Chang, Seokwoo Moon

**Affiliations:** ^1^ Kyungpook National University Hospital, JungGu, Daegu Korea, Republic of (South); ^2^ konkuk University Hospital, Chungju‐SI Korea, Republic of (South)

## Abstract

**Background:**

The Mini‐Mental State Examination (MMSE) is a widely used cognitive screening tool across clinical and research settings. Cognitive performance on the MMSE may be influenced not only by neurodegenerative diseases but also by psychiatric conditions such as depression. This study aims to investigate whether depression affects MMSE item responses and specific cognitive domains using Item Response Theory (IRT) and Differential Item Functioning (DIF) analysis.

**Method:**

A total of 2,007 individuals participated in a nationwide community‐based epidemiological study. The Korean version of MMSE was administered. Depression assessment was determined using the Korean version of the Composite International Diagnostic Interview (K‐CIDI). DIF was assessed using independent samples t‐tests between depressed and non‐depressed groups. Logistic regression analyses evaluated the association between MMSE cognitive domain scores and depression, controlling for age and education.

**Result:**

Significant DIF was observed in multiple MMSE items, especially in domains related to time orientation, registration, attention/calculation, and recall. The IRT model provided detailed psychometric properties, revealing variations in item discrimination and difficulty between groups. Volcano plot analysis revealed strong group differences in these cognitive areas. Significant DIF was observed across several domains, notably time orientation, registration, attention/calculation, and recall, suggesting that depressive symptoms differentially impact cognitive test performance. Logistic regression analyses indicated that deficits in registration (p = 0.027) and attention/calculation (p = 0.074) domains were significantly associated with depression, even after controlling for age and educational attainment. Educational attainment itself was also inversely associated with depression risk (p = 0.046), while age did not show a significant independent effect.

**Conclusion:**

Depression influences specific cognitive domains of the MMSE, particularly registration and attention/calculation abilities. Adjusting for age and education reveals the robust association between these cognitive deficits and depression. The use of IRT methods enhances the understanding of how depressive symptoms affect cognitive test performance at the item level. Incorporating mood assessments when interpreting cognitive test results can enhance diagnostic accuracy and improve individualized treatment planning.